# Isolated right ventricular hypoplasia caused by giant aneurysm of right coronary artery to left ventricle fistula in an adult: a case report

**DOI:** 10.1186/s13019-016-0494-z

**Published:** 2016-07-04

**Authors:** Fawang Zhu, Zhelan Zheng, Lei Yao, Yun Mou, Yan Cheng, Huanhuan Gao

**Affiliations:** Echocardiography and Vascular Ultrasound Center, The First Affiliated Hospital, College of Medicine, Zhejiang University, 79 Qingchun Road, Hangzhou, 310003 Zhejiang China

**Keywords:** Right coronary artery fistula, Right ventricular hypoplasia, Left ventricle, Echocardiography, Cardiac computed tomography angiography, Coronary artery angiography

## Abstract

**Background:**

Right ventricular hypoplasia (RVH) is often caused by tricuspid valve atresia and pulmonary valve atresia. this condition leads to low right ventricular blood volume and right ventricular maldevelopment. But, in adults, the main cause of RVH may also be associated with alloplasia of the right coronary artery, which results in an insufficient blood supply to the right ventricular myocardium. Isolated RVH caused by a right coronary artery fistula is very rare and requires immediate treatment.

**Case presentation:**

We herein report a case involving a 45-year-old man who presented with isolated RVH caused by a giant aneurysm from the right coronary artery to a left ventricle fistula. Echocardiography showed that the right coronary artery was extremely tortuous and obviously dilated with a huge aneurysm. A fistula drained from the right coronary artery into the left ventricle. Moreover, the right heart chamber was significantly collapsed due to extrinsic compression of multiple tortuous, dilated vascular structures. The patient was referred to cardiac surgery. The giant aneurysm was resected, and the proximal and distal openings were closed directly. The fistula was also closed directly, and bypasses were constructed sequentially from the ascending aorta to three branches of the right coronary artery.

**Conclusions:**

Although standard therapeutic strategies of isolated RVH secondary to a right coronary artery fistula are not well established because of the rarity of this condition, our clinical results show that diagnostic echocardiography, coronary artery angiography, and cardiac computed tomography angiography followed by surgical treatment may be an effective management option.

## Background

A coronary artery fistula (CAF) is defined as an anomalous communication between the coronary artery and a cardiac chamber or great vessel. The estimated incidence of CAF is 0.4 % among individuals with congenital heart disease or roughly 1 in 50,000 individuals in the general population [1, 2]. The fistula usually drains into the venous structures of the circulation, such as the right heart chambers, pulmonary artery, coronary sinus and superior vena cava. Drainage into the left ventricle (LV) is less frequent (3 % of CAFs) [1].

Isolated right ventricular hypoplasia (RVH) is a rare congenital heart disease. Its main feature is the absence of the trabecular portion of the right ventricle (RV) with the presence of normally developed tricuspid and pulmonary valves. Only a few case reports and case series describing isolated RVH have been published to date [3–5].

Early death can occur in serious cases of either isolated RVH or CAF [1–4]. However the combination of these two malformations is rarely reported. We herein present a case of isolated RVH secondary to a CAF in an adult with a giant right coronary artery (RCA) aneurysm draining into the LV.

## Case presentation

This study was approved by the Institutional Review Board at the First Affiliated Hospital, College of Medicine, Zhejiang University. The procedures were conducted according to the principles of the Helsinki Declaration.

**Fig. 1:**
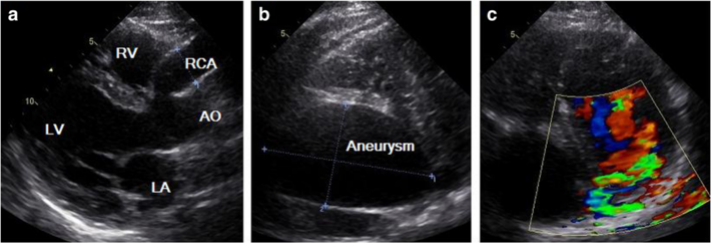
Transthoracic echocardiography (**a**) The obviously dilated RCA, the diameter of which was 2.2 cm. **b** The giant aneurysm measuring 10.0 × 6.1 cm. **c** The fistula draining into the LV through the posterior annulus of the mitral valve

**Fig. 2:**
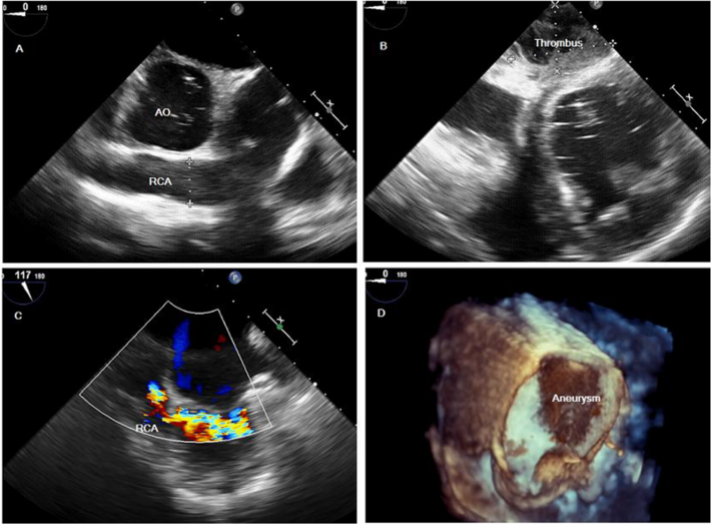
Real-time three-dimensional echocardiography (**a**) The dilated RCA (**b**) Thrombus in the aneurysm, which measured 4.4 × 2.8 cm (**c**) Extremely tortuous RCA draining into the LV through the inferoposterior wall of the LV (**d**) Three-dimensional image of the aneurysm

**Fig. 3:**
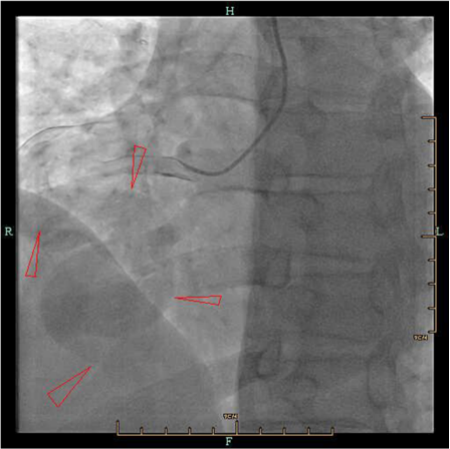
Coronary artery angiography. Contrast agent quickly drained into the LV through the tortuous and dilated RCA

**Fig. 4:**
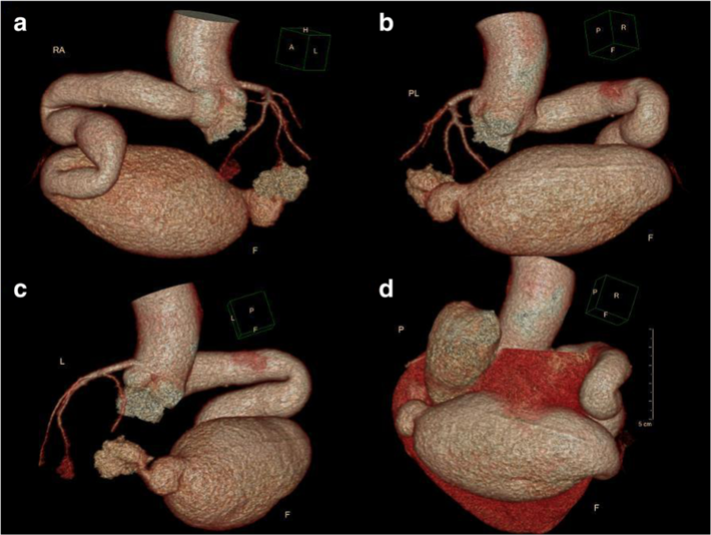
Coronary computed tomography angiography. Serpentine RCA and the giant saccular aneurysm located in the distal portions of the RCA, the diffusely dilated fistula drained into the LV: (**a**) left-anterior oblique view, (**b**) right-posterior oblique view, and (**c**) posteroanterior view. **d** The giant saccular aneurysm (up to 6.1 cm) surrounded the RV

A 45-year-old Chinese man with a 15-day history of worsening intermittent chest congestion was referred to our institution. The patient had no precordium pain; dyspnea; cardiovascular risk factors such as hypertension, diabetes, hyperlipidemia, or kidney disease; or remarkable family history. Physical examination revealed unnoticeable distention of the neck veins, clear consciousness, a heart rate of 65 beats/min with regular rhythm and no murmur, and a blood pressure of 106/75 mmHg (1 mmHg = 0.133 kPa). A 12-lead surface electrocardiogram was normal. A chest X-ray showed significant cardiomegaly with a bulging contour over the right cardiac border and increased bronchovascular markings. Preoperative transthoracic echocardiography (Vivid E9; GE Healthcare, Standpromenaden, Horten, Norway) revealed an extremely RCA. The RCA arose from the right aortic sinus with an opening of about 1.2 cm in diameter. It then became obviously dilated, forming a huge aneurysm (10.0 × 6.1 cm) that extended downward and toward the right on the surface of the RV; it then turned toward the posterior aspect of the heart and formed a fistula draining into the LV through the posterior annulus of the mitral valve. The orifice of the fistula was 0.8 cm in diameter. The maximal velocity of the fistula was approximately 1.8 m/s in the diastolic phase (Fig. 1). Intraoperative transesophageal real-time three-dimensional echocardiography (iE Elite, Philips Healthcare, Bothell, WA, USA) revealed findings similar to those of transthoracic echocardiography: the extremely tortuous RCA drained into the LV through the inferoposterior wall of the LV, and a thrombosis (4.4 cm × 2.8 cm) was present in the aneurysm. Moreover, the right heart chamber was significantly collapsed due to extrinsic compression of multiple tortuous dilated vascular structures with internal blood flows disclosed by color Doppler imaging surrounding the right ventricular myocardium (Fig. 2). Selective coronary artery angiography (CAA) showed that the inner diameter of the RCA was widened, the contrast agent quickly drained into the LV through the tortuous and dilated RCA, the initial diameter of the left coronary artery (LCA) was about 0.58 cm, and there was no collateral branch supplying the RV (Fig. 3). Cardiac computed tomography angiography (CCTA) revealed the whole serpentine RCA, the giant saccular aneurysm (up to 6.1 cm) located in the distal portion of the RCA, and the diffusely dilated fistula draining into the right atrium and then into the LV (Fig. 4).

**Fig. 5:**
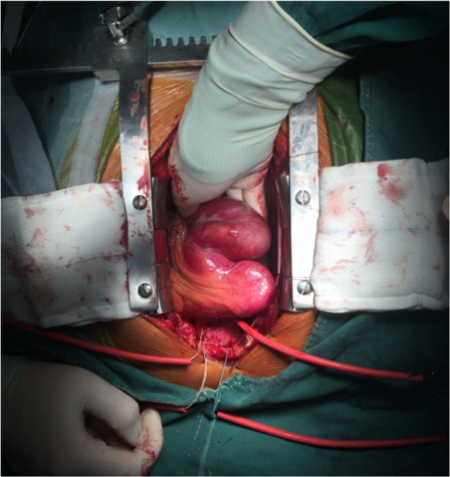
Giant saccular aneurysm of the RCA (approximately 10 × 6 cm) as viewed intraoperatively

Operative findings showed atrophy of the right heart and malformation of the RCA. The trunk, had a diameter of 2 cm and stretched tortuously, giving rise to three branches draining into the RV and LV and finally forming a fistula that drained into the LV. A giant aneurysm measuring approximately 10 × 6 cm was present in front of the fistula orifice, and a thrombus was present within it (Fig. 5). The RCA was closed with sutures up to the orifice of the fistula draining into the LV. Bypasses were constructed sequentially from the ascending aorta to the three branches of the RCA. After resuscitation, the right heart became swollen, and the heart exhibited poor pumping function. Considering that the ostium of the right ventricular branch of the RCA was too small for adequate blood flow, a bypass was reconstructed from the original vein graft to the right ventricular branch of the RCA. The patient almost died of postoperative ventricular fibrillation and congestive heart failure 3 h after surgery; however, he recovered and was discharged from the hospital after 1 month of active treatment.

## Discussions

The clinical manifestations of a CAF generally depend on the hemodynamic and anatomic significance of the lesion. Potential complications of a giant aneurysmal dilated CAF include spontaneous rupture, myocardial ischemia, thrombosis and thromboembolic events. Symptoms arise from the coronary steal phenomenon and diastolic overload [6, 7]. The natural history of CAF development is variable. Symptoms and complications develop with increasing age and depend on the location and size of the fistula; patients can also be asymptomatic during their entire life [2, 7].

Isolated RVH characterized by underdevelopment of the trabecular musculature and a small inner RV diameter, unassociated with severe pulmonary or tricuspid valvular malformation, is a rare condition. The clinical spectrum may vary considerably from severe cyanosis, congestive heart failure, and death in early infancy to mild cyanosis depending on the degree of hypoplasia and the size of the interatrial communication [3–5].

In the present case, the blood volume of the RV was low because the CAF drains into the LV. Part of the LV output is therefore unable to return to the right side of the heart, thus causing an insufficient blood volume in the RV. On the other hand, the RCA, which *de facto* provides a blood supply to the RV wall, is very thin. Furthermore, no obvious collateral artery travels from the LCA to the RV. Therefore, the blood supply of the RV wall is small, leading to a chronically insufficient blood supply to the myocardium of the RV. We consider that the most probable cause of the isolate RVH in the present case was the above-described type of insufficient blood supply and the collapsed RV due to compression by giant aneurysm.

Echocardiography is effective for evaluation of the size and function of the cardiac chambers, the presence and extent of coronary artery dilation, the flow velocity and flow rate of the fistula export, the degree of valvular regurgitation and the pulmonary arterial pressure. We diagnosed CAF in this case based on the presence of dilated and tortuous coronary arteries, and we further evaluate the chamber size and cardiac function using echocardiography. However, the complete anatomy of a CAF is best visualized by CAA and CCTA. These imaging modalities are especially useful in evaluating the features of a CAF, along with important findings such as the origin and drainage sites of the fistula, the serpiginous course of the coronary arteries and their relationship with structures associated with other congenital anomalies, and the presence and severity of atherosclerosis [2, 7, 8].

In the present case, however CAA could not clearly show the coronary artery, which supplies the RV; additionally the orifice, quantity, shape and size of the RV were unclear. The diameter of the LCA was 0.58 cm, and the embranchment of the LCA supplying the RV was not discovered; therefore, the surgeon could not assess blood supply of the RV preoperatively. Because the coronary artery aneurysm was giant, the contrast agent was largely shunted to the LV, and therefore severely diluted within the coronary artery. The orifice of the RCA to the RV was small, and the blood flow rate was low. The RCA embranchment could not be shown, which caused great difficulty during the operation. Because the blood supply of the patient’s RV was completely unknown before the operation, the surgeons needed to determine the location of the supplying arteries by carefully exploring the RV and reconstructing the blood supply channels.

The best therapy for a CAF remains controversial. Surgical closure of a CAF may benefit patients with a large, hemodynamically significant CAF [9]. Our patient undergone surgical treatment, and almost died of postoperative ventricular fibrillation and congestive heart failure. Fortunately, he recovered and was discharged from the hospital after 1 month of active treatment. We consider that most of the blood in the RCA drains into the LV, and that there is normally no LCA embranchment supplying the right ventricular myocardium, This abnormality was the cause of the chronically insufficient myocardial blood flow to the RV that led to RVH in this patient. The preoperative right ventricular dysfunction was relieved because of Zugleichsein of CAF to LV and RVH by steal phenomenon. After the operation, the blood in the RCA no longer drained into the left ventricular chamber, and the RV was no longer compressed by the giant aneurysm. As a result, all of the blood from the systemic circulation drained into the hypogenetic RV, causing right ventricular congestive heart failure. In addition, the patient was treated with oral anticoagulant drugs (aspirin and clopidogrel) for 3 months postoperatively, then only with aspirin. In our opinion, the postoperative ventricular fibrillation and congestive heart failure may have been related to the tiny thrombus.

## Conclusion

In summary, isolated RVH secondary to a giant RCA aneurysm draining into the LV is rare, and echocardiography is helpful for its diagnosis. It is very important to evaluate the size and function of the RV before surgery, and a suitable surgical approach should be chosen to achieve a better therapeutic effect according to the imaging data obtained by echocardiography and radiography. Our clinical results indicate that echocardiography, CCA, and CCTA are useful imaging modalities and that surgical treatment is an effective choice.

## Abbreviations

CAA, coronary artery angiography; CAF, coronary artery fistula; CCTA, cardiac computed tomography angiography; LCA, left coronary artery; LV, left ventricle; RCA, right coronary artery; RV, right ventricle; RVH, right ventricular hypoplasia.
